# Impact of green/blue spaces on specific morbidity and cause-specific mortality in Belgium: the GRESP-HEALTH project protocol (2015-2019)

**DOI:** 10.1186/2049-3258-73-S1-P16

**Published:** 2015-09-17

**Authors:** Lidia Casas, Isabelle Thomas, Tim Nawrot, Catherine Bouland, Patrick Deboosere, An Van Nieuwenhuyse, Benoit Nemery

**Affiliations:** 1 KU Leuven, Leuven, Belgium; 2 UCL, Louvain-la-Neuve, Belgium; 3 U Hasselt, Hasselt, Belgium; 4 ULB, Brussels, Belgium; 5 VUB, Brussels, Belgium; 6 WIV, Brussels, Belgium

## 

Living in green/blue areas is associated with better health. This may be due to low air and/or noise pollution, opportunities for physical activity, facilitation of social contacts, and promotion of recovery from fatigue and stress. Yet, socio-economic (SE) factors also explain inequalities in health and access to green/blue spaces. The GRESP-HEALTH project evaluates the associations between living in/close to a green/blue area on morbidity and mortality in Belgium. It assesses all-cause and cause-specific mortality, specific morbidities and perceived health, considering environmental pollutants and SE factors.

The project includes individuals registered in the Belgian censuses of 1991 and/or 2001. Three levels of observation are studied: individual, statistical sector (SS) and group of SS, following individual and ecological designs. Mortality information is based on the National Mortality Database (a linkage between cause-specific mortality (2001-2010), perceived health (2001 census) and SE factors (1991 and 2001 censuses). Morbidity information (2004-2012) is derived from the IMA (Intermutualistisch Agentschap) database, which contains reimbursement data of prescriptions. For green/blue spaces, the surface, shape, accessibility and type are calculated for each SS. Residential area-specific exposure to air pollutants is obtained from satellite images. Traffic noise databases are used whenever possible. We will consider SE factors such as material deprivation, education, and occupation. The analyses will be conducted separately in different age specific populations and types of area (urban, sub-urban, rural). We will use multilevel models for clustered data within geographical areas. Interactions of green/blue spaces with air pollution and SE factors will be evaluated and stratified analyses in areas with similar SE and environmental characteristics will be performed. Moreover, specific population groups (gender, employment status) will be considered.

The GRESP-HEALTH project will improve the scientific knowledge about the hitherto uncertain associations between living close to green/blue spaces and health.

